# Supplementary material for: Ciprofloxacin Concentrations in Food Could Select for Quinolone Resistance in Klebsiella pneumoniae: An In Vivo Study in Galleria mellonella
Source: Antibiotics (Basel). 2024 Nov 18;13(11):1097. doi: 10.3390/antibiotics13111097 (PMC11591287; doi:10.3390/antibiotics13111097)
Supplement: Supplementary file 1 [file antibiotics-13-01097-s001.zip › antibiotics-3264651-supplementary.pdf]

Supplementary

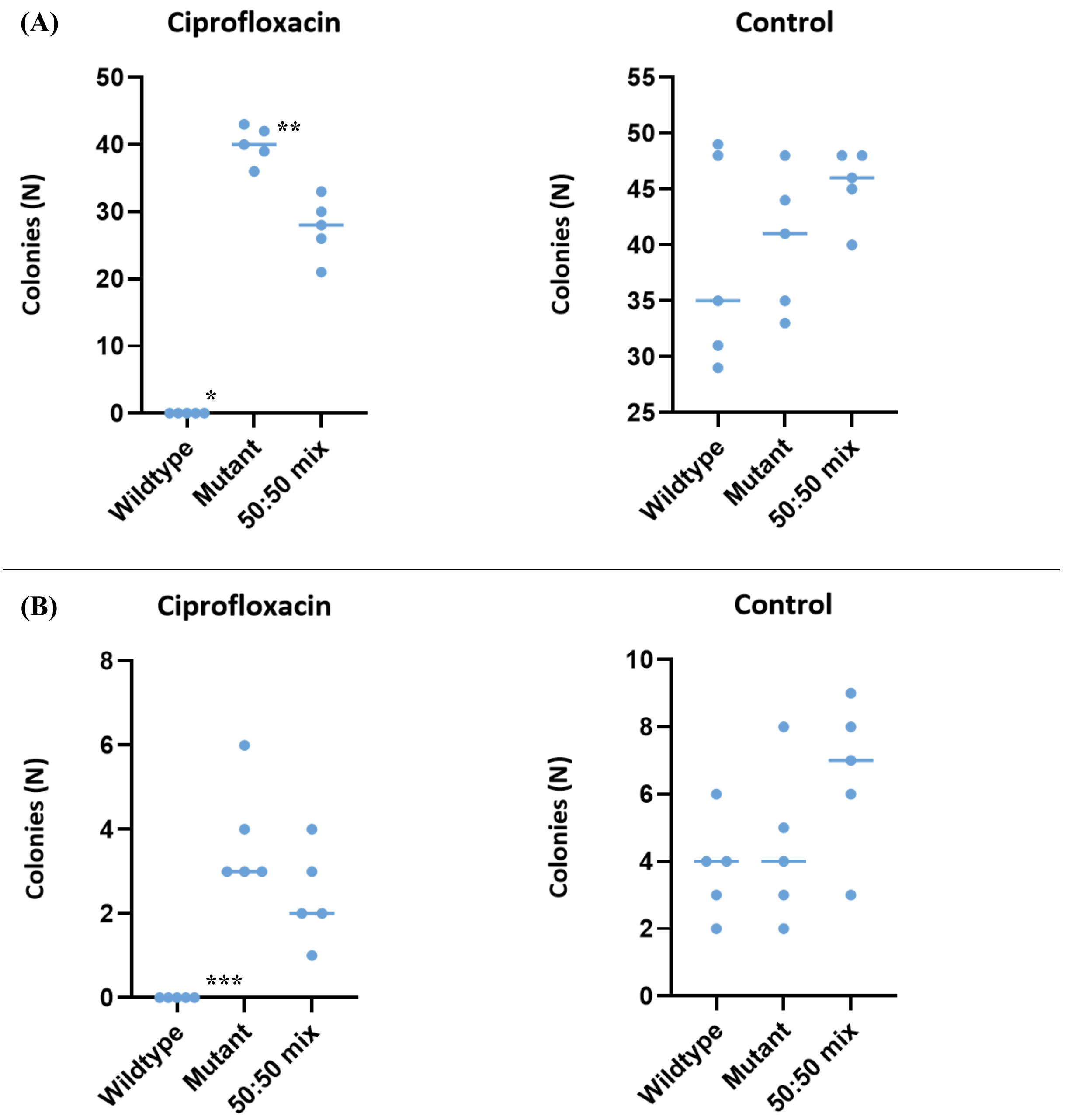

Figure S1: Scatterplots showing the validation of the 50:50 ratio of the *K. pneumoniae* mix using a serial dilution passage of concentrations 10^{-5} and 10^{-6}. (A) The colonies (N) were automatically counted with the colony counter for concentration 10^{-5} on plates with ciprofloxacin and without ciprofloxacin (control). (B) The number of colonies (N) was counted on plates with and without ciprofloxacin (control) for concentrations 10^{-6}. The lines represent the median. P-values were considered significant below 0.05. (A) The wildtype (*) and mutant (**) strains showed a P-value of 0.0079 for the ciprofloxacin plates. (B) Only the wildtype strain showed a significant P-value of 0.0079 (***) for the ciprofloxacin plates.

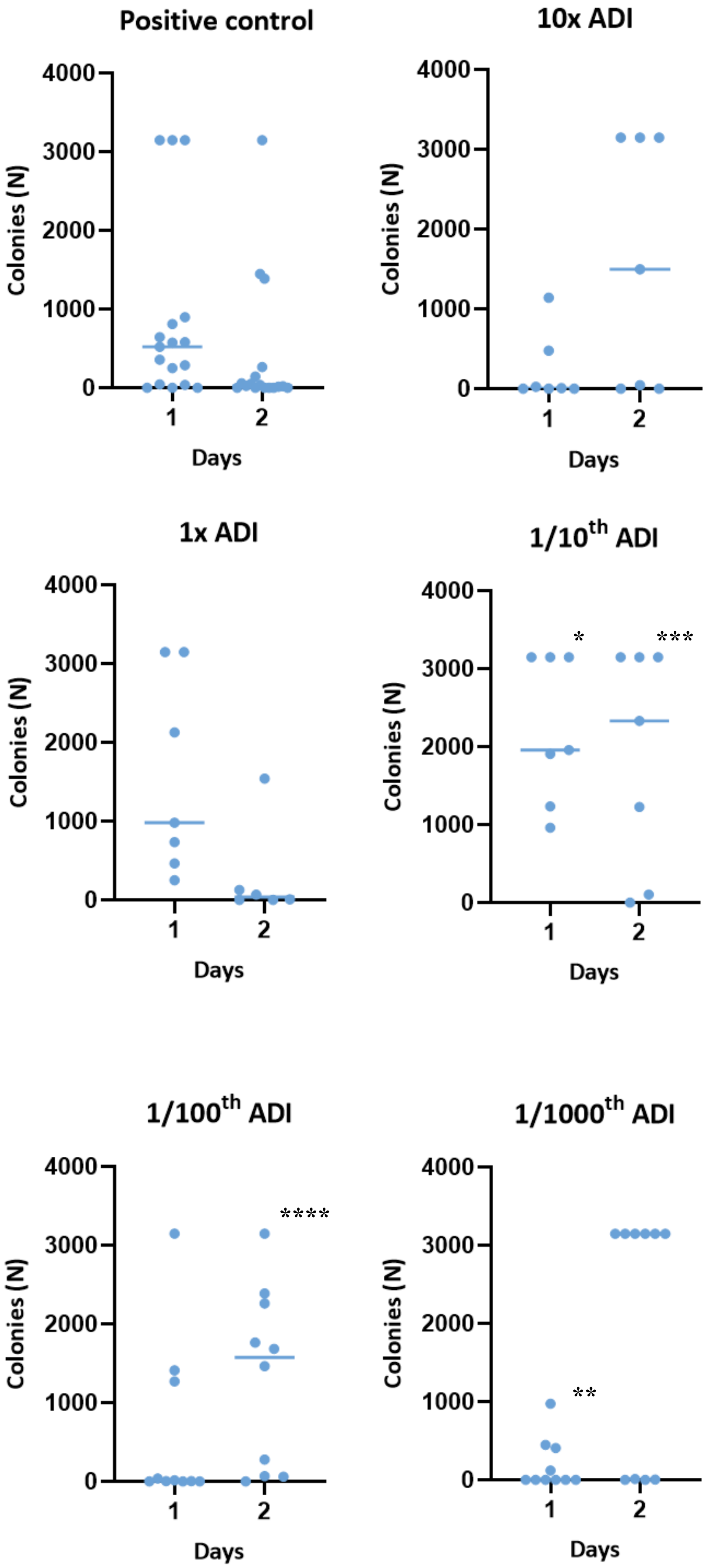

Positive control
10x ADI
1x ADI
1/10th ADI
1/100th ADI
1/1000th ADI
Colonies (N)
Days
0
1000
2000
3000
4000
1
2
*
**
***
****

Figure S2: Scatterplots showing the success of colonization of *G. mellonella* with *K. pneumoniae* mix followed by various concentrations of ciprofloxacin (10x ADI, 1x ADI, 1/10th, 1/100th and 1/1000th ADI) or PBS (positive control). The number of colonies that appeared were counted from control plates without ciprofloxacin (control) after 24 and 48 hours. Colony count of the colonies of the larvae containing the *K. pneumoniae* mix together with PBS and of larvae injected with different ADI doses of ciprofloxacin: 23.9 ng/µL (10x ADI), 2.39 ng/µL (1x ADI), 0.239 ng/µL (1/10th ADI), 0.00239 ng/µL (1/100th ADI) and 0.000239 ng/µL (1/1000th ADI). The lines represent the median. P-values were considered significant <0,05. Two significant values were seen for day 1 at 1/10th concentration (P = 0.0040*) and 1/1000th concentration (P = 0.0366**). For day 2, two significant values were seen at 1/10th concentration (P = 0.0378***) and 1/100th concentration (P = 0.0105****).

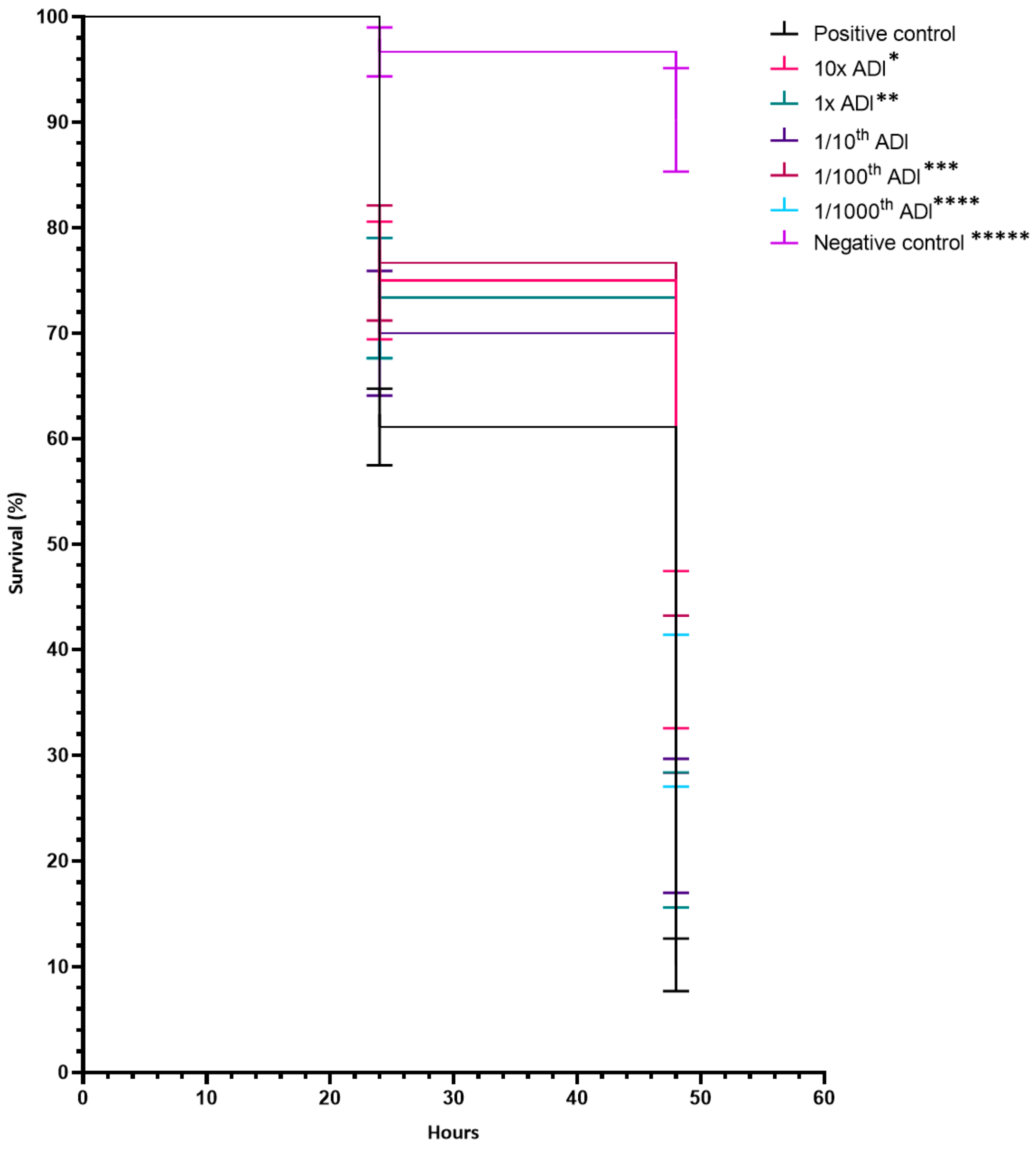

Figure S3: A Kaplan-Meier survival curve of *Galleria mellonella* larvae injected with *Klebsiella pneumoniae* mix alone (positive control) and together with different ADI concentrations of ciprofloxacin. The P-values were retrieved comparing each different ciprofloxacin group to the positive control group using a Gehan-Breslow-Wilcoxon test. P-values were considered significant below 0.05. The bars represent the standard error (SE). The asterisks represent different P-values compared to the positive control group: (*) 0.0021; (**) 0.0373; (***) 0.0020; (****) 0.0087; (*****) <0.0001.

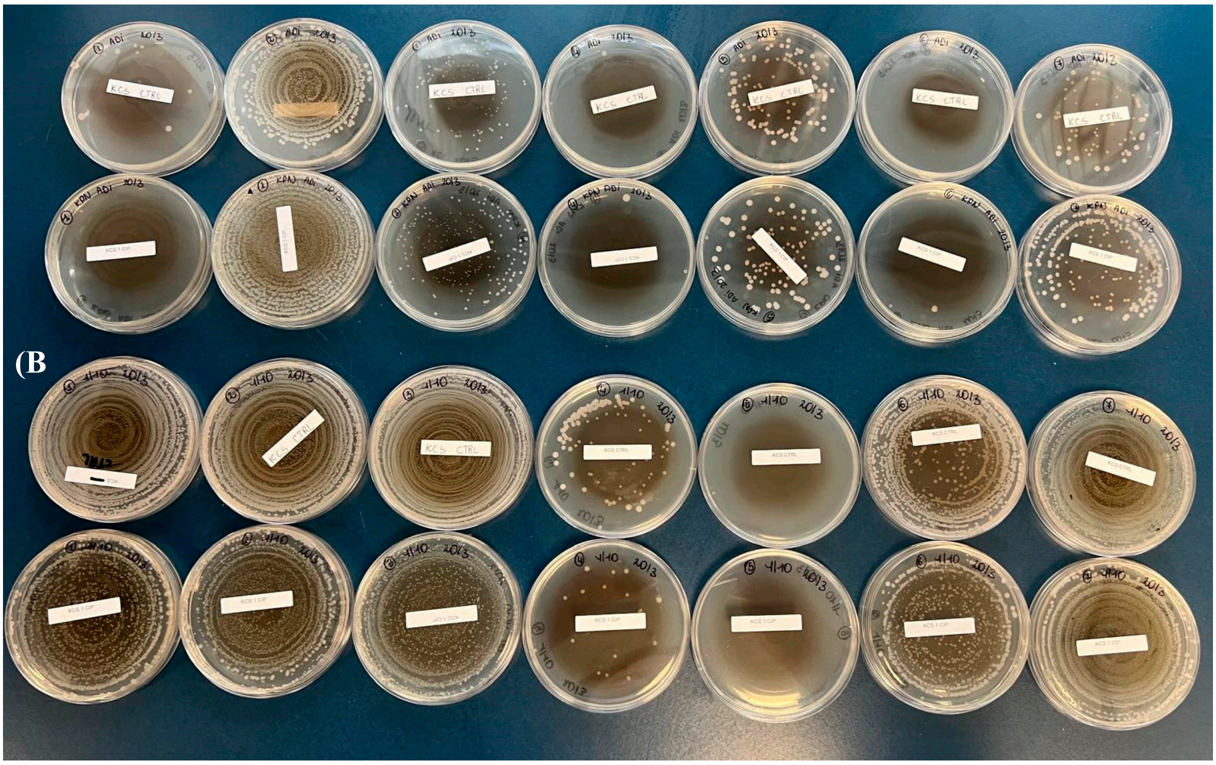

Figure S4: Visualization of the plates plated after hemolymph extraction for the 1x ADI and 1/10th ADI ciprofloxacin concentration on day 1. (A) Hemolymph containing *K. pneumoniae* mix and 1x ADI ciprofloxacin concentration plated out on control (first row) and ciprofloxacin (second row) plates. (B) Hemolymph containing *K. pneumoniae* mix and 1/10th ciprofloxacin concentration plated out on control (first row) and ciprofloxacin (second row) plates. Each pair of plates, control and ciprofloxacin, is from one independent larvae.

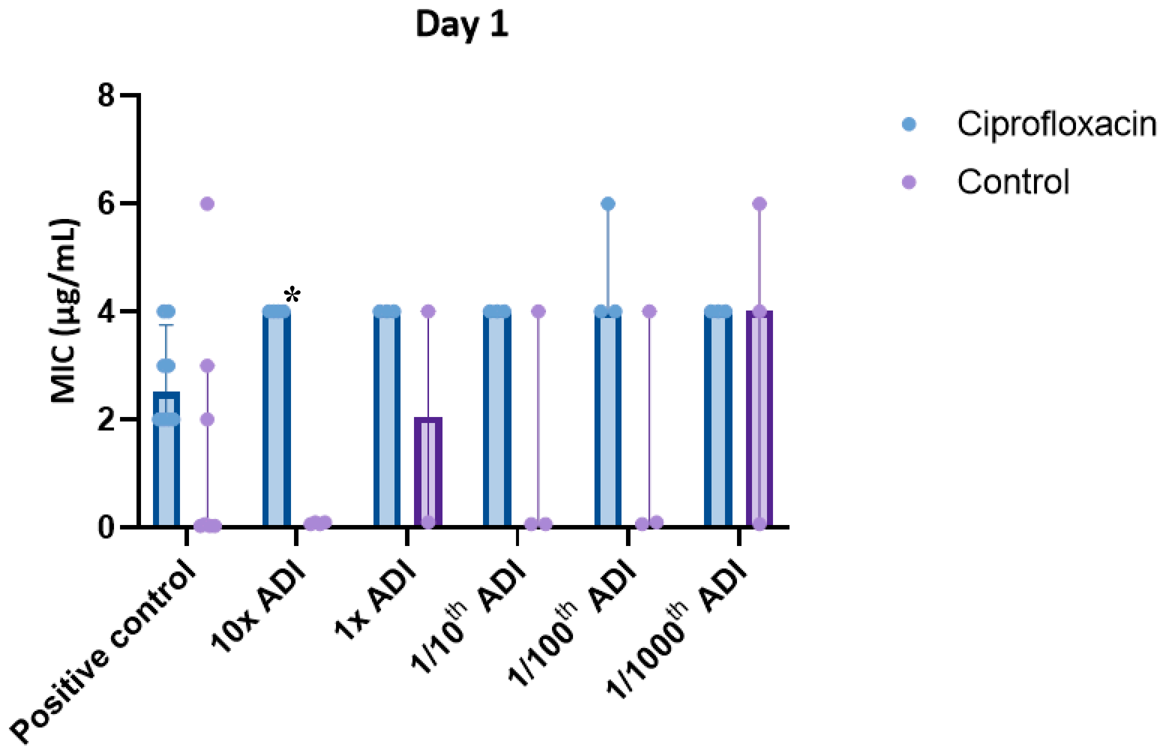

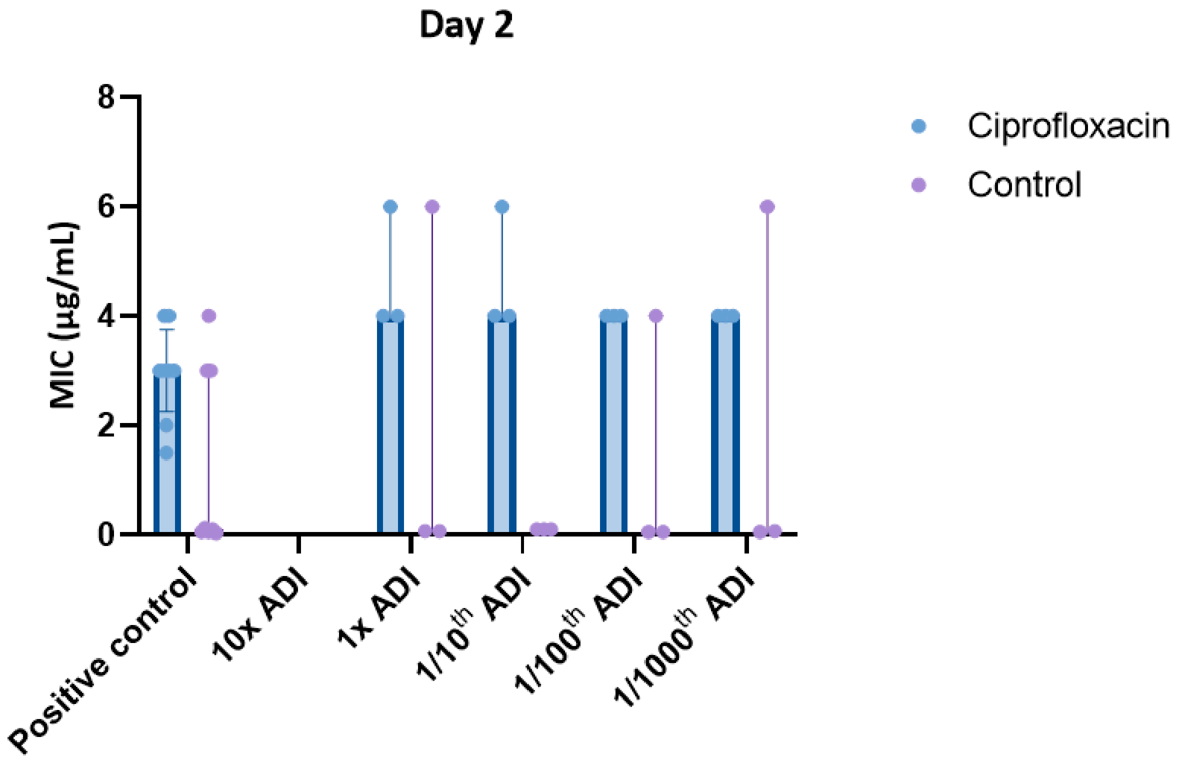

Figure S5: MIC (µg/mL) distribution of the plates with ciprofloxacin and without ciprofloxacin (control) for day 1 and day 2. The distribution is shown for all the ADI concentrations and the positive control. The bars represent the interquartile range and the line shows the median. The asterisk shows a significant P-value of 0.0485 for 10x ADI concentration from the ciprofloxacin plates compared to the positive control.

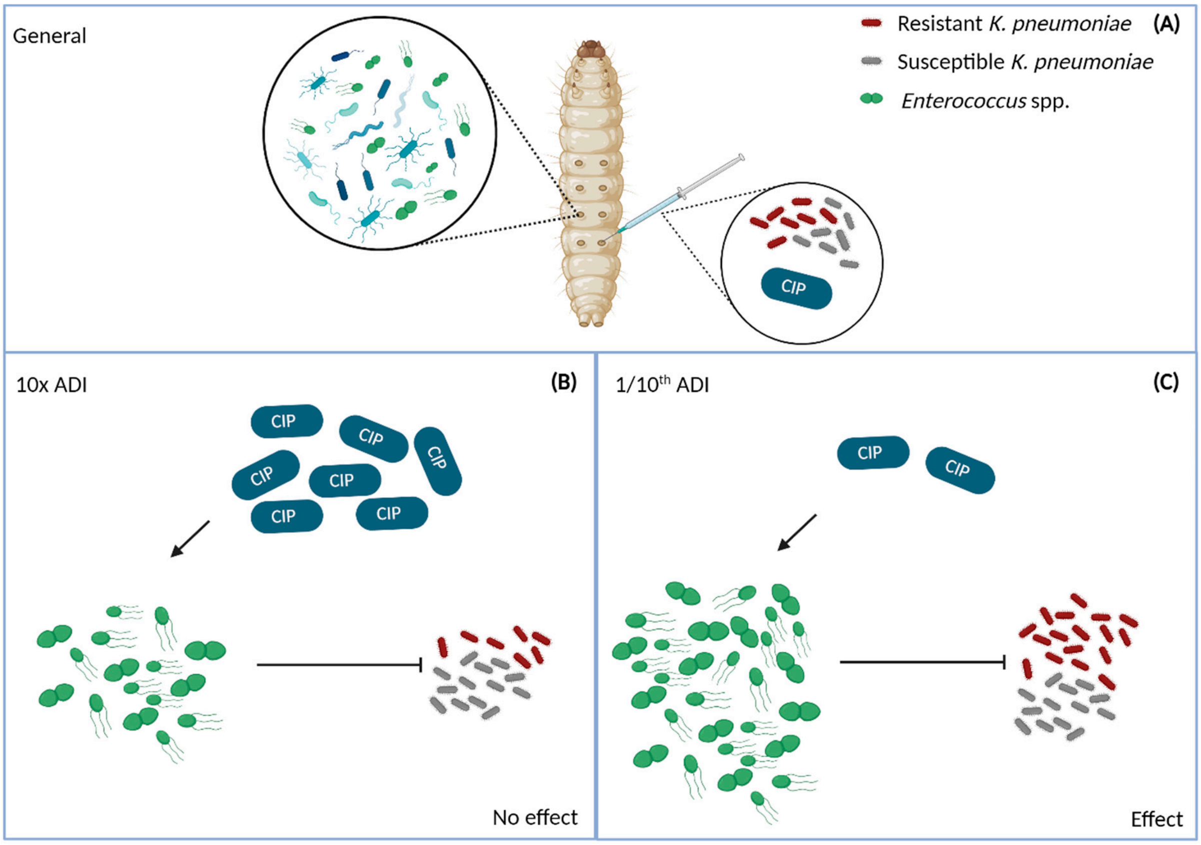

Figure S6: Depiction of one hypothesized effect of the *G. mellonella* microbiome on the ratio of ciprofloxacin-resistant to susceptible strains of *K. pneumoniae* at different ciprofloxacin concentrations. The selective selection of resistant strains of *K. pneumoniae* at 1x ADI and 1/10th ADI of ciprofloxacin concentrations could be explained as follows. *Enterococcus* spp. is the dominant bacterial species in the microbiome of *Galleria mellonella*, while *K. pneumoniae* is not naturally present within this microbiome (A). Upon injecting *K. pneumoniae* into the wax moth larvae, it can colonize the larvae, but it could be inhibited by *Enterococcus faecalis* (Figure 15b). A study has found that *E. faecalis* can inhibit *K. pneumoniae* by producing lactic acid. Thus, *E. faecalis* could inhibit the growth of both the wildtype and the mutant strains of *K. pneumoniae*. Other bacterial species could have similar effects and may inhibit the susceptible and resistant strains of *K. pneumoniae* to a different extent. Antibiotics have been shown to lead to dysbiosis within the larvae. Ciprofloxacin is bacteriocidal against Gram-positive and Gram-negative bacteria, including the *Enterococcus* spp. and *Klebsiella pneumonia*. Thus, differential ciprofloxacin-induced changes in the microbiota at different concentrations of ciprofloxacin could result in different relative growth rates for the susceptible and resistant isolates (B and C).

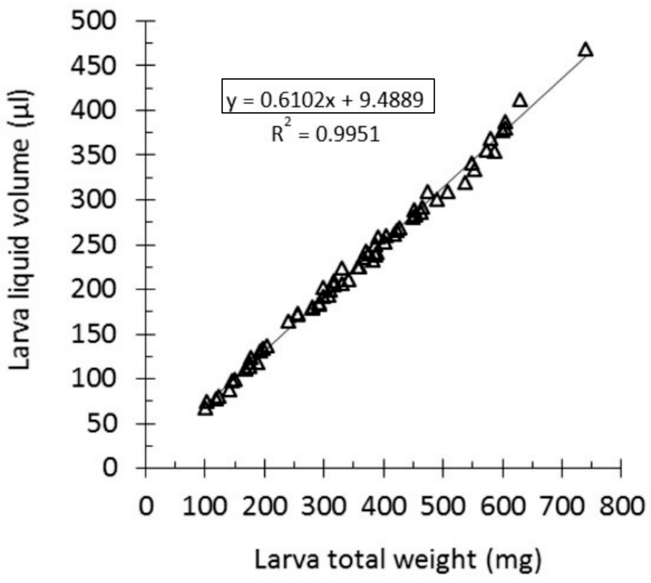

Figure S7: The larva liquid volume (µL) in function of the larva total weight (mg). The larva's liquid weight was determined to be the wet-minus-dry weight, which was almost similar to the liquid volume. Data per single larva is indicated with a triangle. The R square was calculated in Excel. [Figure and caption adapted from Andrea et al.]

Table S1: Overview of the required ciprofloxacin concentrations injected in the larvae for each different ADI condition. The required concentration was estimated with this average weight according to the formulas of Andrea et al. Milli Q water was used to dilute the ciprofloxacin concentrations.

**10x ADI (23.9 ng/µL)**				
Average weight larvae (x)		366.31 mg		
Required concentration of ciprofloxacin ($C_{compound}$)		0.064 µg		
C1	V1	C2	V2	Milli Q
10 µg	64 µL	0.64 µg	1500 µL	936 µL
**1x ADI (2.39 ng/µL) and 1/10th ADI (0.239 ng/µL)**				
Average weight larvae (x)		416.31 mg		
Required concentration of ciprofloxacin ($C_{compound}$)		0.07 µg		
C1	V1	C2	V2	Milli Q
0.2 µg	358 µL	0.07 µg	1500 µL	642 µL
0.2 µg	35.8 µL	0.007 µg	1500 µL	964.2 µL
**1/100th ADI (0.00239 ng/µL) and 1/1000th ADI (0.000239 ng/µL)**				
Average weight larvae (x)		402.97 mg		
Required concentration of ciprofloxacin ($C_{compound}$)		0.0697 µg		
C1	V1	C2	V2	Milli Q
0.2 µg	7 µL	0.000697 µg	2000 µL	1993 µL
0.05 µg	2.8 µL	0.0000697 µg	2000 µL	1997.2 µL

Table S2: The colony counts of the positive control (*K. pneumoniae* mix and PBS) and the different ciprofloxacin ADI concentrations on the ciprofloxacin (CIP) and control (CTRL) plates together with their different proportions and median proportions. The proportions were calculated with the formula (ciprofloxacin/control).

PBS (24h Ext)	CIP	CTRL	Proportion	PBS(48h Ext)	CIP	CTRL	Proportion
Positive control 1	357	289	1.235294118	Positive control 1	0	1	0
Positive control 2	385	644	0.597826087	Positive control 2	41	16	2.5625
Positive control 3	0	576	0	Positive control 3	0	37	0
Positive control 4	62	39	1.58974359	Positive control 4	0	1	0
Positive control 5	25	810	0.030864198	Positive control 5	3147	3147	
Positive control 6	318	522	0.609195402	Positive control 6	0	2	0
Positive control 7	3147	3147		Positive control 7	0	0	
Positive control 8	3147	3147		Positive control 8	41	146	0.280821918
Positive control 9	388	583	0.665523156	Positive control 9	205	265	0.773584906
Positive control 10	250	251	0.996015936	Positive control 10	1	2	0.5
Positive control 11	0	0		Positive control 11	756	1447	0.522460263
Positive control 12	0	46	0	Positive control 12	0	59	0
Positive control 13	0	0		Positive control 13	26	50	0.52
Positive control 14	736	899	0.81868743	Positive control 14	0	1	0
Positive control 15	0	357	0	Positive control 15	0	22	0
Positive control 16	419	3147	0.133142676	Positive control 16	3	25	0.12
Positive control 17	0	0		Positive control 17	77	1388	0.055475504
Median	250	522	0.603510745	Median	1	25	0.055475504
**23.9ng/μL (24h Ext)**	**CIP**	**CTRL**	**Proportion**	**23.9ng/μL (48h Ext)**	**CIP**	**CTRL**	**Proportion**
L1 10x ADI	0	1	0	L1 10x ADI	1	0	
L3 10x ADI	1	0		L2 10x ADI	0	0	
L4 10x ADI	25	479	0.052192067	L3 10x ADI	3147	3147	
L5 10x ADI	27	24	1.125	L4 10x ADI	1	3147	0.000317763
L6 10x ADI	11	9	1.222222222	L5 10x ADI	1312	1497	0.876419506
L7 10x ADI	637	1140	0.55877193	L6 10x ADI	122	46	2.652173913
L8 10x ADI	0	0		L7 10x ADI	3147	3147	
Median	11	9	0.55877193	Median	122	1497	0.876419506
**2.39ng/μL (24h Ext)**	**CIP**	**CTRL**	**Proportion**	**2.39ng/μL (48h Ext)**	**CIP**	**CTRL**	**Proportion**
L1 1x ADI	1853	2128	0.870770677	L1 1x ADI	2	9	0.222222222
L2 1x ADI	50	982	0.050916497	L2 1x ADI	2358	1540	1.531168831
L3 1x ADI	23	253	0.090909091	L3 1x ADI	1	2	0.5
L4 1x ADI	364	466	0.78111588	L4 1x ADI	114	129	0.88372093
L5 1x ADI	3147	3147		L5 1x ADI	1	0	
L6 1x ADI	3147	3147		L6 1x ADI	283	68	4.161764706
L7 1x ADI	655	735	0.891156463				
Median	655	982	0.78111588	Median	58	38.5	0.88372093
**0.239ng/μL (24h Ext)**	**CIP**	**CTRL**	**Proportion**	**0.239ng/μL (48h Ext)**	**CIP**	**CTRL**	**Proportion**
L1 1/10th ADI	3147	3147		L1 1/10th ADI	1317	2331	0.564993565
L2 1/10th ADI	535	962	0.556133056	L2 1/10th ADI	1902	3147	0.604385129
L3 1/10th ADI	3147	1234	2.550243112	L3 1/10th ADI	1926	3147	0.612011439
L4 1/10th ADI	800	1959	0.408371618	L4 1/10th ADI	17	105	0.161904762
L5 1/10th ADI	3147	3147		L5 1/10th ADI	0	0	
L6 1/10th ADI	3147	3147		L6 1/10th ADI	863	1229	0.702196908
L7 1/10th ADI	973	1908	0.509958071	L7 1/10th ADI	2569	3147	0.816333016
Median	3147	1959	0.533045564	Median	1317	2331	0.608198284
**.00239ng/μL (24h Ext)**	**CIP**	**CTRL**	**Proportion**	**0.00239ng/μL (48h Ext)**	**CIP**	**CTRL**	**Proportion**
L1 1/100th ADI	0	5	0	L1 1/100th ADI	1543	1765	0.874220963
L2 1/100th ADI	0	0		L2 1/100th ADI	0	67	0
L3 1/100th ADI	0	2	0	L3 1/100th ADI	3147	3147	
L4 1/100th ADI	6	39	0.153846154	L4 1/100th ADI	2	60	0.033333333
L5 1/100th ADI	263	1413	0.186128804	L5 1/100th ADI	44	2260	0.019469027
L6 1/100th ADI	4	6	0.666666667	L6 1/100th ADI	0	0	
L7 1/100th ADI	3147	3147		L7 1/100th ADI	1009	2388	0.422529313
L8 1/100th ADI	0	15	0	L8 1/100th ADI	189	1686	0.112099644
L9 1/100th ADI	873	1270	0.687401575	L9 1/100th ADI	0	1464	0
L10 1/100th ADI	0	0		L10 1/100th ADI	198	277	0.714801444
Median	2	10.5	0.153846154	Median	116.5	1575	0.072716489
**000239ng/μL (24h Ext)**	**CIP**	**CTRL**	**Proportion**	**0.000239ng/μL (48h Ext)**	**CIP**	**CTRL**	**Proportion**
L1 1/1000th ADI	0	0		L1 1/1000th ADI	0	11	0
L2 1/1000th ADI	0	0		L2 1/1000th ADI	0	6	0
L3 1/1000th ADI	242	447	0.541387025	L3 1/1000th ADI	3147	3147	
L4 1/1000th ADI	0	0		L4 1/1000th ADI	0	1	0
L5 1/1000th ADI	0	121	0	L5 1/1000th ADI	3147	3147	
L6 1/1000th ADI	0	0		L6 1/1000th ADI	0	3147	0
L7 1/1000th ADI	413	407	1.014742015	L7 1/1000th ADI	3147	3147	
L8 1/1000th ADI	366	976	0.375	L8 1/1000th ADI	3147	3147	
L9 1/1000th ADI	1	0		L9 1/1000th ADI	401	3147	0.127422942
L10 1/1000th ADI	0	0		L10 1/1000th ADI	0	3	0
Median	0	0	0.458193512	Median	200.5	3147	0

Table S3: Statistical analyses of the proportions of different ciprofloxacin ADI concentrations compared to the positive control. The Mann-Whitney test was performed with the different ciprofloxacin ADI concentrations against the positive control. Two significant values were found for the 1x ADI (P = 0.0233) and the 1/10th ADI (P = 0.0162). The Kruskal-Wallis test was used to test the overall difference between all the different groups. A significant P-value of 0,0070 < 0,05 was found for the proportions of day 2. The two-stage linear step-up of the Benjamini test was used to adjust for multiple comparisons.

24 hours extraction (day 1)						
Mann-Whitney test			Significant	P-value		
Positive control vs. 10x			No	0.8224		
Positive control vs. 1x			No	0.5727		
Positive control vs. 1/10th			No	0.7703		
Positive control vs. 1/100th			No	0.3448		
Positive control vs. 1/1000th			No	0.8940		
Kruskal-Wallis test						
P-value	0.6878 > 0.05					
Two-stage linear step-up of Benjamini			Significant	q value		Individual P-value
Positive control vs. 10x			No	0.9158		0.7305
Positive control vs. 1x			No	0.9158		0.6160
Positive control vs. 1/10th			No	0.9158		0.5332
Positive control vs. 1/100th			No	0.9158		0.2602
Positive control vs. 1/1000th			No	0.9158		0.8722
Interquartile range (IQR)						
Positive control	1/1000th ADI	1/100th ADI	1/10th ADI	1x ADI	10x ADI	
0.007716	0.09375	0	0.4338	0.07091	0.0261	Q1
0.6035	0.4582	0.1538	0.533	0.7811	0.5588	Median
0.8127	0.8964	0.6667	2052	1	1.174	Q3
**48 hours extraction (day 2)**						
Mann-Whitney test			Significant	P-value		
Positive control vs. 10x			No	0.1066		
Positive control vs. 1x			Yes	0.0233		
Positive control vs. 1/10th			Yes	0.0162		
Positive control vs. 1/100th			No	0.7198		
Positive control vs. 1/1000th			No	0.1869		
Kruskal-Wallis test						
P-value	0,0070 < 0,05					
Two-stage linear step-up of Benjamini			Significant	q value		Individual P-value
Positive control vs. 10x			No	0.1487		0.0850

Positive control vs. 1x	No	0.0596	0.0113
Positive control vs. 1/10th	No	0.0975	0.0371
Positive control vs. 1/100th	No	0.7407	0.7054
Positive control vs. 1/1000th	No	0.2698	0.2056

Interquartile range (IQR)

Positive control	1/1000th ADI	1/100th ADI	1/10th ADI	1x ADI	10x ADI	
0	0	0.004867	0.4642	0.3611	0.000318	Q1
0.12	0	0.07272	0.6082	0.8837	0.8764	Median
0.5225	0.06371	0.6417	0.7307	2.846	2.652	Q3

Table S4: MIC (µg/mL) and identification for each different colony on the ciprofloxacin and control plates that were picked.

Positive control (24h Ext)	MIC (CIP) µg/mL	Identification	MIC (Control) µg/mL	Identification	Positive control (48h Ext)	MIC (CIP) µg/mL	Identification	MIC (Control) µg/mL	Identification
Positive control 1	3	*K. pneumoniae*	0,032	*K. pneumoniae*	Positive control 2	2	*K. pneumoniae*	0,047	*K. pneumoniae*
Positive control 2	2	*K. pneumoniae*	3	*K. pneumoniae*	Positive control 5	3	*K. pneumoniae*	0,047	*K. pneumoniae*
Positive control 4	3	*K. pneumoniae*	0,064	*K. pneumoniae*	Positive control 2	3	*K. pneumoniae*	3	*K. pneumoniae*
Positive control 2	2	*K. pneumoniae*	2	*K. pneumoniae*	Positive control 3	3	*K. pneumoniae*	3	*K. pneumoniae*
Positive control 3	2	*K. pneumoniae*	0,032	*K. pneumoniae*	Positive control 4	1,5	*K. pneumoniae*	0,032	*K. pneumoniae*
Positive control 4	2	*K. pneumoniae*	0,032	*K. pneumoniae*	Positive control 3	4	*K. pneumoniae*	4	*K. pneumoniae*
**23.9 ng/µL (24h Ext)**	**MIC (CIP) µg/mL**	**Identification**	**MIC (Control) µg/mL**	**Identification**	**23.9 ng/µL (48h Ext)**	**MIC (CIP) µg/mL**	**Identification**	**MIC (Control) µg/mL**	**Identification**
L3 10x ADI	4	*K. pneumoniae*	0,064	*K. pneumoniae*					
L5 10x ADI	4	*K. pneumoniae*	0,094	*K. pneumoniae*					
L6 10x ADI	4	*K. pneumoniae*	0,064	*K. pneumoniae*					
L7 10x ADI	4	*K. pneumoniae*	0,094	*K. pneumoniae*					
**2.39 ng/µL (24h Ext)**	**MIC (CIP) µg/mL**	**Identification**	**MIC (Control) µg/mL**	**Identification**	**2.39 ng/µL (48h Ext)**	**MIC (CIP) µg/mL**	**Identification**	**MIC (Control) µg/mL**	**Identification**
L2 1x ADI	4	*K. pneumoniae*	Mutant and susceptible	*K. pneumoniae*	L4 1x ADI	6	*K. pneumoniae*	6	*K. pneumoniae*
L3 1x ADI	4	*K. pneumoniae*	4	*K. pneumoniae*	L5 1x ADI	4	*K. pneumoniae*	0,064	*K. pneumoniae*
L4 1x ADI	4	*K. pneumoniae*	0,094	*K. pneumoniae*	L7 1x ADI	4	*K. pneumoniae*	0,064	*K. pneumoniae*
**0.239 ng/µL (24h Ext)**	**MIC (CIP) µg/mL**	**Identification**	**MIC (Control) µg/mL**	**Identification**	**0.239 ng/µL (48h Ext)**	**MIC (CIP) µg/mL**	**Identification**	**MIC (Control) µg/mL**	**Identification**
L2 1/10th ADI	4	*K. pneumoniae*	0,064	*K. pneumoniae*	L2 1/10th ADI	4	*K. pneumoniae*	0,094	*K. pneumoniae*
L3 1/10th ADI	4	*K. pneumoniae*	0,064	*K. pneumoniae*	L4 1/10th ADI	4	*K. pneumoniae*	0,094	*K. pneumoniae*
L7 1/10th ADI	4	*K. pneumoniae*	4	*K. pneumoniae*	L6 1/10th ADI	6	*K. pneumoniae*	0,094	*K. pneumoniae*
**0.00239 ng/µL (24h Ext)**	**MIC (CIP) µg/mL**	**Identification**	**MIC (Control) µg/mL**	**Identification**	**0.00239 ng/µL (48h Ext)**	**MIC (CIP) µg/mL**	**Identification**	**MIC (Control) µg/mL**	**Identification**
L4 1/100th ADI	6	*K. pneumoniae*	0,064	*K. pneumoniae*	L4 1/100th ADI	4	*K. pneumoniae*	0,047	*K. pneumoniae*
L5 1/100th ADI	4	*K. pneumoniae*	0,094	*K. pneumoniae*	L5 1/100th ADI	4	*K. pneumoniae*	0,047	*K. pneumoniae*
L9 1/100th ADI	4	*K. pneumoniae*	4	*K. pneumoniae*	L10 1/100th ADI	4	*K. pneumoniae*	4	*K. pneumoniae*
**0.000239 ng/µL (24h Ext)**	**MIC (CIP) µg/mL**	**Identification**	**MIC (Control) µg/mL**	**Identification**	**0.000239 ng/µL (48h Ext)**	**MIC (CIP) µg/mL**	**Identification**	**MIC (Control) µg/mL**	**Identification**
L3 1/1000th ADI	4	*K. pneumoniae*	4	*K. pneumoniae*	L3 1/1000th ADI	4	*K. pneumoniae*	6	*K. pneumoniae*
L7 1/1000th ADI	4	*K. pneumoniae*	6	*K. pneumoniae*	L8 1/1000th ADI	4	*K. pneumoniae*	0,064	*K. pneumoniae*
L8 1/1000th ADI	4	*K. pneumoniae*	0,064	*K. pneumoniae*	L9 1/1000th ADI	4	*K. pneumoniae*	0,047	*K. pneumoniae*

Table S5: The meaning of the reliability values of MALDI-TOF MS Bruker [Figure from].

Meaning of score value

Rang	Description	Symbols	Color
2.300...3.000	Highly probable species identification	(+++)	Green
2.000...2.299	Secure genus identification, probable species identification	(++)	Green
1.700...1.999	Probable genus identification	(+)	Yellow
0.000...1.699	Not reliable identification	(-)	Red
